# Management of rituximab in patients with PLA2R-associated membranous nephropathy of different age: a single-center retrospective cohort study

**DOI:** 10.1080/0886022X.2025.2555688

**Published:** 2025-09-07

**Authors:** Xueting Li, Lei Song, Le Wang, Ben Ke, Wen Shen

**Affiliations:** ^a^ Department of Nephrology, The Second Affiliated Hospital of Nanchang University, Nanchang, China; ^b^ Department of General Medicine, The Second Affiliated Hospital of Nanchang University, Nanchang, China; ^c^ Department of Blood Transfusion, The Second Affiliated Hospital to Nanchang University, Nanchang, China; ^d^ Department of Nephrology, The Second Affiliated Hospital of Nanchang University, Jiangxi Province Key Laboratory of Immunology and Inflammation, The Second Affiliated Hospital, Jiangxi Medical College, Nanchang University, Nanchang, China; ^e^ Department of Cardiovascular Medicine, The Second Affiliated Hospital of Nanchang University, Nanchang, China

**Keywords:** Idiopathic membranous nephropathy, older, low-dose and long-course rituximab regimen, infection, eosinophilic granulocytes

## Abstract

**Background:**

Rituximab (RTX) has become the first-line therapy for idiopathic membranous nephropathy (IMN). The safety of low-dose and long-course RTX regimen in elderly patients with IMN remains unknown.

**Methods:**

Sixty-nine IMN patients with anti-M-phospholipase A2 receptor (PLA2R) antibodies-positive were recruited for this study. The patients were categorized into two groups based on their age. The different age groups were further divided into the recommended RTX group and the low-dose and long-course RTX group. Compare the outcomes and adverse events of patients between different groups after 9-month follow-up.

**Results:**

There was no significant difference in the complete remission rate and composite remission rate in patients with IMN in different age who received different RTX regimens. As expected, the risk of adverse events was higher in the recommended-dose RTX group compared with the low-dose and long-course RTX group in patients with IMN aged ≥60 years (66.7% vs. 19%, *p* = .006), and the main adverse event was infection (*p* = .019). Moreover, we found that different regimens were independent risk factor for infection in patients with IMN aged ≥60 years. Furtherly, ROC curve analysis suggest that in the first month after RTX used, compared with the percentage of CD19^+^ B lymphocytes, the percentage of eosinophilic granulocytes was more sensitive in predicting the risk of infection in elderly IMN patients (AUC = 0.329 vs. AUC = 0.555).

**Conclusions:**

The low-dose and long-course RTX regimen should be recommended for elderly patients with IMN, and the percentage of eosinophilic granulocytes is a better risk predictor of infections after RTX used in elderly patients with IMN.

## 1. Introduction

Idiopathic membranous nephropathy (IMN) is an autoimmune kidney disease, and the treatment of this disease has made great progress in recent years [1]. In 2009, a ground-breaking study identified that M-phospholipase A2 receptor (PLA2R) as the primary target antigen in IMN [2]. Anti-PLA2R antibody is not only associated with disease activity, but also significantly associated with the prognosis of IMN [3–5].

According to the guidelines for the diagnosis and treatment of glomerulonephritis issued by KDIGO in 2021, in patients with anti-phospholipase A2 receptor (PLA2R) antibody-positive membranous nephropathy, if the clinical manifestations are typical, kidney biopsy may not be required to confirm the diagnosis [6].

In the past decade, the treatment of IMN has made great progress [7]. Rituximab (RTX) is a selective B-cell depleting drug, which has become the first-line therapy for IMN [8]. Moreover, increasing studies have confirmed that the efficacy of RTX is as well as traditional treatments, and RTX therapy has a more favorable safety profile [9–12]. However, some evidence suggested that the recommended RTX may contribute a great risk of infection in older patients with IMN [13–15]. Our previous study showed that optimized RTX regimen was as effective as the recommended regimen in patients with IMN and had fewer side effects [15]. In this retrospective study, 69 eligible IMN patients with anti-PLA2R antibodies-positive were enrolled and divided into two groups based on age. The clinical remission rate, including complete response rate and partial response rate, and adverse event in these patients were explored.

## 2. Methods

### 2.1. Study patients

**Figure 1.**
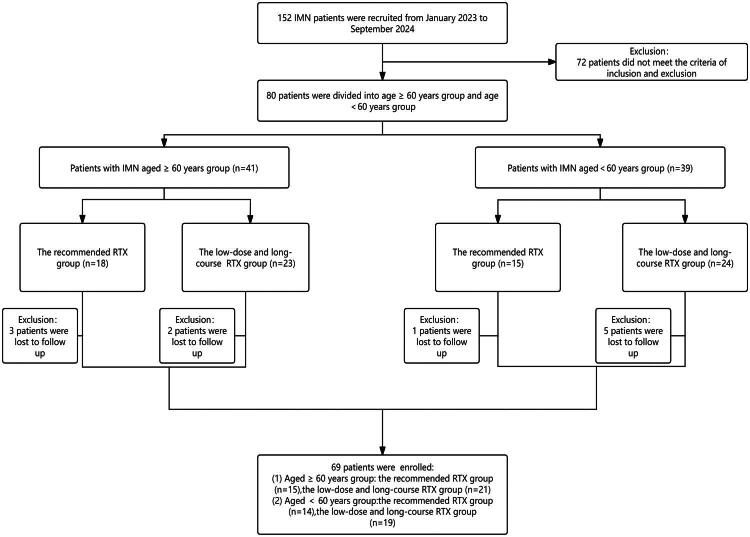
Flowchart of the study.

This study is a single-center retrospective analysis. From January 2023 to September 2024, 152 IMN patients with anti-PLA2R antibodies-positive confirmed by kidney biopsy were recruited from the Department of Nephrology, The Second Affiliated Hospital of Nanchang University (Figure 1). This study was conducted in strict accordance with the Declaration of Helsinki and was approved by the Ethics Committee of the Second Affiliated Hospital of Nanchang University (No. 20220114C). All patients signed the informed written consent. Inclusion criteria: (i) patients diagnosed with IMN by kidney biopsy, and anti-phospholipase-A2 receptor (anti-PLA2R) antibody: >20 RU/mL; (ii) patients met the diagnostic criteria for nephrotic syndrome; (iii) no treatment with glucocorticoids or immunosuppressants before ­kidney biopsy.

Exclusion criteria: (i) patients with type 1 or type 2 diabetes; (ii) secondary MN; (iii) the presence of active infection, or pregnancy or breastfeeding; (iv) having received glucocorticoids or immunosuppressants therapy within the last 3 months; (v) severe liver dysfunction or cardiovascular disease.

### 2.2. Intervention

According to the guideline recommendations [8], the recommended RTX regimen involved an infusion of 1,000 mg on day 1 and day 15 and the low-dose and long-course RTX regimen was 500 mg each time, once a week, for a course of 4 consecutive weeks.

### 2.3. Data collection and follow-up

Patients’ medical information, such as age, biologic sex, blood pressure, albuminuria, serum albumin (ALB), creatinine, eGFR, and anti-PLA2R antibody titer data, was collected. Serum levels of anti-PLA2R antibodies were evaluated using an ELISA kit (Euroimmune, Lubeck, Germany). All basic tests were completed before immunotherapy. All severe adverse reactions were treated and recorded both in hospitalization and follow-up.

Follow up will be conducted on all patients at 3, 6, and 9 months after treatment to collect relevant clinical data.

### 2.4. Outcomes

The primary clinical outcome was composite remission, which was defined as complete remission (CR) or partial remission (PR) within 9 months. CR: urinary protein excretion ≤ 0.3g/d; ALB ≥ 35g/L. PR: urinary protein < 3.5 g/d, 50% lower than baseline; ALB ≥ 35g/L. Composite remission comprises CR or PR.

The severe adverse events were documented, such as infection, leukopenia, and diabetes as secondary clinical outcomes.

### 2.5. Statistical analysis

Nonnormally distributed data are represented by the median (Q25 and Q75), and continuous variables are compared using the Wilcoxon rank sum test. Normal distribution data are represented by mean ± SD, and continuous variable comparison is performed using *t*-test. Categorical data are represented by counts and percentages, while nominal variables are compared using Chi-square tests. The cumulative response rate was calculated using Kaplan–Meier’s method and evaluated using log rank method. Using logistic regression analysis, the factors associated with adverse events were explored. The sensitivity and specificity of relevant indicators were evaluated using ROC curve. Double tailed *p* < .05 is considered statistically significant. SPSS version 22.0 (IBM SPSS Inc., Chicago, IL) was used to analysis all data.

## 3. Results

### 3.1. Study participants

Sixty-nine IMN patients were recruited for this study, of whom 36 patients were aged ≥60 years and 33 patients were aged <60 years. In the patients aged ≥60 years, 15 patients received the recommended-dose of RTX regimen, and 21 patients received a low-dose and long-course RTX regimen. In patients aged <60 years, 14 patients received the recommended-dose RTX regimen and 19 patients received a low-dose and long-course RTX regimen.

All clinical indices at baseline included age, sex, blood pressure, albuminuria, ALB, serum creatinine, anti-PLA2R antibody titer, and eGFR. These data were similar among different groups (Table 1).

**Table 1.** Baseline characteristics of IMN patients in different age treated with different doses of RTX regimens.

Characteristics	IMN patients aged ≥60 years			IMN patients aged <60 years		
Groups	Recommended RTX group (*n* = 15)	Low-dose and long-course RTX group (*n* = 21)	*p*	Recommended RTX group (*n* = 14)	Low-dose and long-course RTX group (*n* = 19)	*p*
Age (years)	66 (63, 70)	71 (65, 75)	.059	48 (44, 55)	52 (37, 56)	.715
Anti-PLA2R antibody titer (RU/mL)	315.81 (106.72, 457.14)	108.82 (60.48, 147.61)	.056	106.21 (71.58, 219.78)	144.35 (35.03, 249.18)	.884
Sex, *n* (%)			.175			.510
Males	8 (53.3%)	16 (76.2%)		10 (76.9%)	15 (78.6%)	
Females	7 (46.7%)	5 (23.8%)		3 (23.1%)	3 (21.1%)	
BP (mmHg)						
SBP	121.34 ± 14.61	128.07 ± 16.76	.064	120.64 ± 14.77	122.16 ± 15.50	.773
DBP	73.67 ± 11.61	79.43 ± 11.02	.140	76.29 ± 8.22	79.16 ± 13.15	.478
Proteinuria (mg/24 h)	9,005.26 (5,383.03, 12,392.22)	9,636.20 (7,47.47, 13,489.80)	.413	10,302.37 (8,206.26, 13,864.44)	8,846.70 (6,394.83, 12,675.00)	.274
Albumin (g/L)	25.70 (20.83, 28.01)	24.86 (19.58, 27.51)	.385	22.79 (19.61, 25.81)	24.10 (21.10, 26.40)	.551
Serum creatinine (μmol/L)	82.70 (64.20, 96.80)	97.20 (76.35, 130.86)	.127	88.70 (79.19, 108.05)	88.31 (69.08, 99.74)	.489
eGFR (mL/min/1.73 m^2^)	80.67 (65.62, 87.35)	68.93 (45.15, 89.02)	.242	88.50 (59.96, 101.47)	83.32 (77.29, 99.38)	.743
Number of CD19^+^ B lymphocytes	268.55 ± 138.92	162.00 ± 43.89	.092	180.00 ± 62.33	152.69 ± 117.90	.536
Proportion of CD19^+^ B lymphocytes	13.94 ± 6.08	13.26 ± 4.00	.768	12.63 ± 3.39	11.80 ± 6.94	.299

### 3.2. Primary outcomes comparison among different groups

**Figure 2.**
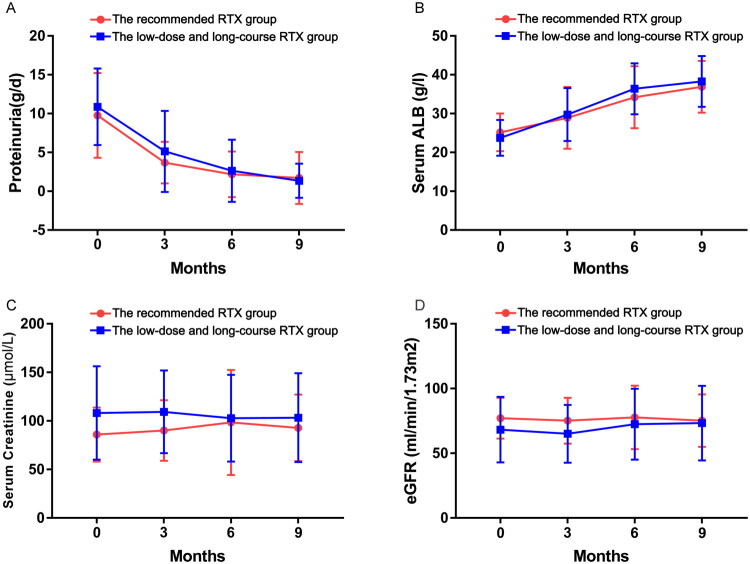
Changes in clinical indicators during follow-up in patients with IMN aged ≥60 years. (A) 24 h proteinuria. (B) Serum albumin. (C) Serum creatinine. (D) Estimated glomerular filtration rate (eGFR).

**Figure 3.**
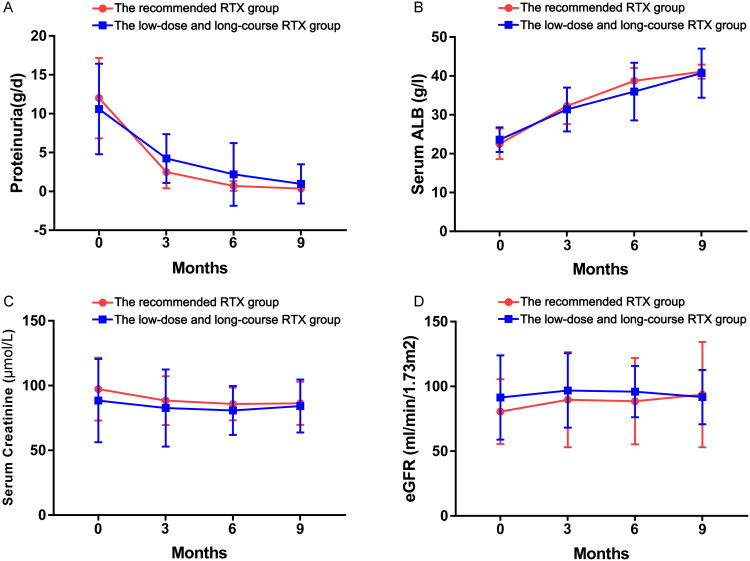
Changes in clinical indicators during follow-up in patients with IMN aged <60 years. (A) 24 h proteinuria. (B) Serum albumin. (C) Serum creatinine. (D) Estimated glomerular filtration rate (eGFR).

Table 2 summarizes the results of the different age groups using different doses of RTX regimens at 9 months of follow-up and the comparison of relevant laboratory indices. The data of the follow-up showed no statistical difference in the complete and composite remission rates between the group receiving the recommended dose of RTX and the group receiving the low-dose and long-course RTX regimen at 9 months, either in patients aged ≥60 years or in patients aged <60 years (aged ≥60 years: *p* = .892, *p* = .650; aged <60 years: *p* = .947, *p* = .823). Moreover, there was no significant difference in the median time to achieve composite remission between the two different age groups using different regimen groups (*p* = .715; *p* = .463). Among patients aged ≥60 years, two patients in recommended RTX group and four patients in low-dose and long-course RTX group did not achieve composite remission. There are no statistical difference in clinical parameters, including 24 h proteinuria, ALB, blood pressure, scr, and eGFR at 9-month follow-up in either patients aged ≥60 years or <60 years (Figures 2 and 3).

**Table 2.** Comparison of results and laboratory indices of IMN patients at 9-month follow-up after treatment with different doses of RTX regimens in different age groups.

Characteristics	Aged ≥60 years group			Aged <60 years group		
Groups	Recommended RTX group (*n* = 15)	Low-dose and long-course RTX group (*n* = 21)	*p*	Recommended RTX group (*n* = 14)	Low-dose and long-course RTX group (*n* = 19)	*p*
Composite remission, *n* (%)	13 (86.7%)	17 (81.0%)	.650	13 (92.9%)	18 (94.7%)	.823
Complete remission, *n* (%)	5 (33.3%)	7 (33.3%)	.892	5 (35.7%)	7 (36.8%)	.947
Median time to composite remission *n* (%)	3.0 (3.0, 6.0)	3.0 (3.0, 6.0)	.715	3.0 (3.0, 6.0)	4.5 (3.0, 6.0)	.463
No-response, *n* (%)	2 (13.3%)	4 (19.0%)	.674	0 (0)	1 (5.3%)	–
BP (mmHg)						
SBP	123 ± 19.88	132.69 ± 12.11	.056	113.64 ± 7.97	119.47 ± 10.51	.092
DBP	75.31 ± 10.21	76.50 ± 7.22	.514	71.36 ± 8.68	76.37 ± 9.08	.121
Proteinuria (mg/24 h)	534.98 (228.38, 1,858.63)	473.91 (243.91, 2,138.04)	.835	382.42 (204.00, 477.01)	369.47 (212.34, 562.17)	.799
Albumin (g/L)	38.90 (31.05, 41.95)	34.14 (39.50, 42.32)	.432	40.71 (39.56, 42.93)	41.78 (38.57, 44.37)	.798
Serum creatinine (μmol/L)	83.50 (70.15, 112.49)	94.35 (73.00, 129.83)	.498	81.27 (70.52, 94.06)	84.01 (65.62, 88.26)	.838
eGFR (mL/min/1.73 m^2^)	77.82 (60.57, 89.28)	73.76 (51.13, 98.05)	.839	97.28 (68.45, 112.25)	90.23 (81.93, 110.26)	.843

**Figure 4.**
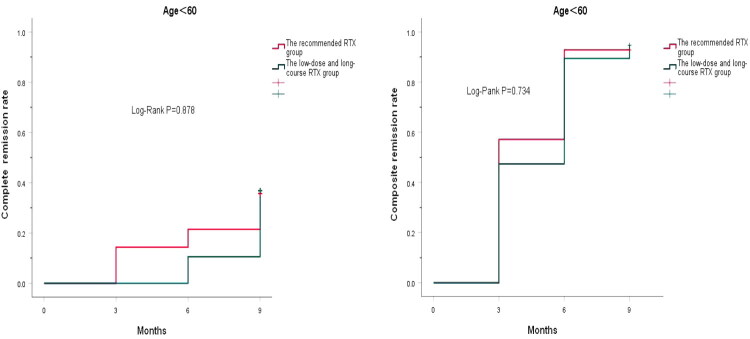
Cumulative complete remission rate and cumulative composite remission rate in the patients with IMN aged ≥60 years during follow-up.

**Figure 5.**
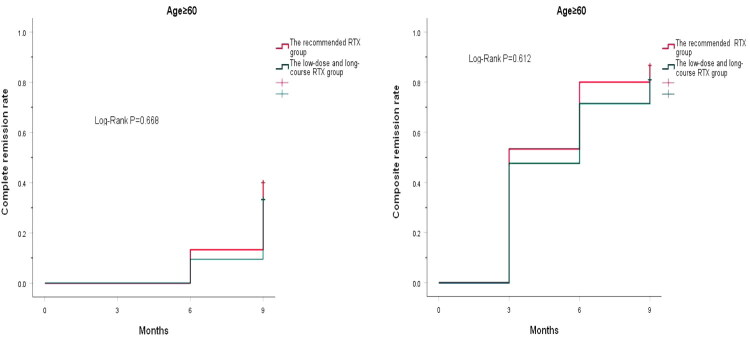
Cumulative complete remission rate and cumulative composite remission rate in the patients with IMN aged <60 years during follow-up.

Furthermore, the cumulative CR rate and the cumulative composite remission rate showed no statistical significance between the recommended RTX group and the low-dose and long-course RTX group at 9 months, either in patients aged ≥60 years or in patients aged <60 years (*p* = .878, *p* = .734, *p* = .668, *p* = .612) (Figures 4 and 5).

### 3.3. Safety evaluation

In Table 3, all the side effects that occurred during the 9-month follow-up in the IMN patients were included in the study. In the age <60 group, only one adverse event occurred in patients receiving the recommended RTX regimen, which was urinary tract infection, and there was no statistical difference between the two regimens. In the age ≥60 years group, 10 patients (66.7%) in recommended RTX group suffered adverse events, four patients (19.0%) in low-dose and long-course RTX group experienced adverse events, and there was a significant difference between the two groups (*p* = .006). Moreover, among the adverse events that occurred in these two groups, the percentage of infection that occurred in the different regimens was the highest, with seven patients (46.7%) in recommended RTX group and two patients (9.5%) in the low-dose and long-course RTX group, which was statistically different (*p* = .019). In addition to infections, one patient experienced leukopenia in the recommended RTX group, and one patient suffered steroidogenic diabetes mellitus and one patient suffered liver function abnormalities in both groups.

**Table 3.** Summary of adverse events at 9-month follow-up after treatment with different doses of RTX regimens in IMN patients at different age.

Groups	Aged ≥60 years group			Aged <60 years group		
Events	Recommended RTX group (*n* = 15)	Low-dose and long-course RTX group (*n* = 21)	*p*	Recommended RTX group (*n* = 14)	Low-dose and long-course RTX group (*n* = 19)	*p*
Any adverse event (*n*)	10 (66.7%)	4 (19%)	.006	1 (7.1%)	0 (0)	.424
Steroid-induced diabetes, *n* (%)	1 (6.7%)	1 (4.8%)		0 (0)	0 (0)	
Leukopenia, *n* (%)	1 (6.7%)	0 (0)	.417	0 (0)	0 (0)	
Infection, *n* (%)	7 (46.7%)	2 (9.5%)	.019	1 (7.1%)	0 (0)	
Pulmonary infection, *n* (%)	6 (40.0%)	2 (9.5%)	.046	0 (0)	0 (0)	
Urinary tract infection, *n* (%)	1 (6.7%)	0 (0)	.417	1 (7%)	0 (0)	.424
Herpes zoster, *n* (%)	0 (0)	0 (0)	–	0 (0)	0 (0)	
Abnormal liver function, *n* (%)	1 (6.7%)	1 (4.8%)				

### 3.4. Post analysis

For all IMN patients included in this study, we used logistic regression analysis to examine the factors associated with the risk of infection after receiving different dose of RTX (Table 4). Univariate regression analysis showed that different RTX treatment protocols (OR 7.238; 95%CI: 1.406, 37.260, *p* = .018) and age (OR 1.074; 95%CI: 1.002, 1.152, *p* = .043) were closely related to the infection. Further multivariate logistic regression analysis showed that different treatment protocols (OR 17.893; 95%CI: 2.360, 135.670, *p* = .005) and age (OR 1.135; 95%CI: 1.025, 1.255, *p* = .014) were independent risk factors for infections in patients with IMN (Table 4).

**Table 4.** Factors associated with the risk of infection at 9-month follow-up after RTX treatment in patients with IMN (logistic regression).

	Univariate regression analysis			Multivariate regression analysis		
Factors	OR	95%CI	*p*	OR	95%CI	*p*
Different treatment protocols	7.238	(1.406, 37.260)	**.018**	14.320	(1.765, 116.166)	**.005**
Sex (female)	0.188	(0.046, 0.769)	.064	0.225	(0.043, 1.168)	.076
Age (increase by 1 year)	1.074	(1.002, 1.152)	**.043**	1.121	(1.016, 1.236)	**.014**
Anti-PLA2R antibody titer baseline value (RU/mL)	1.001	(0.998, 1.004)	.522			
Baseline SBP	1.007	(0.966, 1.049)	.740			
Baseline DBP	0.964	(0.905, 1.027)	.256			
Baseline proteinuria (increase by 1 g/d)	1.000	(0.980, 1.020)	.325			
Baseline Albumin (increase by 1 g/L)	1.122	(0.946, 1.330)	.186			
Baseline serum creatinine (increase by 1 μmol/L)	1.000	(0.982, 1.019)	.997			
Baseline eGFR (increase by 1 mL/min/1.73 m^2^)	0.985	(0.957, 1.013)	.282			
Number of CD19^+^ B lymphocytes	1.299	(0.788, 2.141)	.305			
Proportion of CD19^+^ B lymphocytes	5.676	(0.082, 391.788)	.422			
Percentage of eosinophilic granulocytes	1.003	(0.586, 1.719)	.990			

Note: The data in bold means the different treatment protocols and age among factors associated with the risk of infection are of significant.

Based on these analyses, we further performed logistic regression analyses in patients aged ≥60 years with IMN. The results showed that the different treatment protocols (OR 8.312; 95%CI: 1.408, 49.063, *p* = .019) were independent risk factors for infection in patients with IMN aged ≥60 years (Table 5).

**Table 5.** Factors associated with risk of infection with 9 months in patients aged ≥60 years treated with RTX (logistic regression).

	Univariate regression analysis		
	OR	95%CI	*p*
Different treatment protocols	8.312	(1.408, 49.063)	.019
Age (increase by 1 year)	1.008	(0.889, 1.144)	.897
Anti-PLA2R antibody titer baseline value (RU/mL)	1.001	(0.997, 1.004)	.736

**Figure 6.**
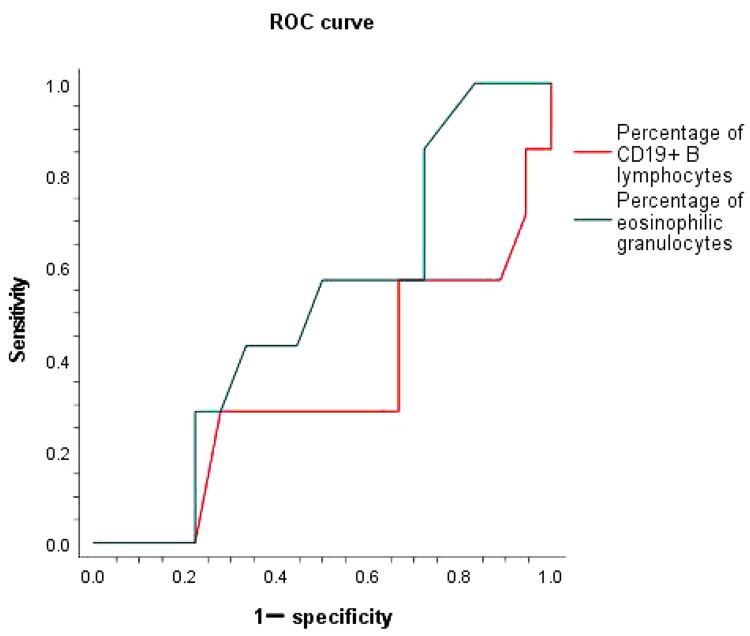
Comparison of the sensitivity of CD19^+^ B lymphocyte percentage and eosinophilic granulocytes percentage to predict infection in patients with IMN who received RTX after 1 month.

It is known that the number of CD20^+^/CD19^+^ B lymphocytes is not only one of the important indicators to judge the therapeutic effect of RTX, but also one of the important indicators to predict the adverse reactions of IMN patients [16,17]. Generally, a lower number of B lymphocytes predicts a higher incidence of adverse reactions in IMN patients after RTX use [16,17]. In this study, we used ROC curve analysis to predict the sensitivity and specificity of infection after RTX treatment in IMN patients aged ≥60 years, based on the percentage of CD19^+^ B lymphocytes. We found that the percentage of CD19^+^ B lymphocyte did not predict the incidence of infection after RTX was used in IMN patients (*p* = .625). However, ROC curve analysis showed that in the first month after RTX was used, compared with the percentage of CD19^+^ B lymphocytes, the percentage of eosinophilic granulocytes was more sensitive in predicting the incidence of infection in elderly patients with IMN (AUC = 0.329 vs. AUC = 0.555), although neither of these two indicators was a good predictor of infection risk (Table 6, Figure 6).

**Table 6.** Comparison of the sensitivity of CD19^+^ B lymphocyte percentage and eosinophilic granulocytes percentage to predict infection in patients with IMN who received RTX after 1 month.

Area under the ROC curve	
Test outcome variables	Area
Percentage of CD19^+^ B lymphocytes	0.329
Percentage of eosinophilic granulocytes	0.555

## 4. Discussion

In recent years, as the prevalence of IMN is increasing, the efficacy of traditional glucocorticoid combined with alkylating agent (cyclophosphamide) or calcineurin inhibitor is not satisfactory in clinical practice. Since 2009, PLA2R1 on glomerular podocytes have been detected in the majority of IMN patients [2], which has led to greater progress in the study of the pathogenesis of IMN.

RTX, the current first-line therapeutic option for IMN, has been preceded by several studies demonstrating its efficacy and safety. For example, the RI-CYCLO trial found that the efficacy and safety of corticosteroid combined cyclophosphamide and RTX were comparable [12]. The results of the MENTOR study also suggested that the relapse rate and efficacy of RTX were significantly lower than that of the cyclosporine group, while the incidence of serious adverse events was slightly lower than that of the cyclosporine group [10]. However, Maxted et al. reported that low-dose RTX treatment for nephrotic syndrome is not effective [18]. Moreover, Moroni at al. also found that low-dose RTX obtains remission in <50% of PMN patients [19]. It is notable that the patients included in the study by Maxted et al. were children with glucocorticoid dependence or frequently relapsing nephrotic syndrome, and the RTX treatment regimen adopted in the study by Moroni et al. was RTX (375 mg/m^2^) once or twice. These two studies were completely different with our study. In the 2021 KDIGO guidelines, the recommended dose of RTX for the treatment of IMN is 1,000 mg on each of days 1 and 15, or 375 mg/m^2^ each time, once a week for 4 consecutive weeks as a course of treatment [8]. In this study, IMN patients who received the RTX regimen recommended by the guidelines were taken as the control group, and IMN patients who received the low-dose, long-course RTX regimen were taken as the intervention group (500 mg each time per week for 4 consecutive weeks). We found that the recommended dose of RTX regimen for elderly IMN patients is not only poorly tolerated, but also has a high incidence of adverse events. Our previous studies have shown that optimized RTX regimens are as effective as recommended RTX regimens in patients with IMN and have fewer side effects, especially in older patients with IMN [15]. In this study, a larger sample size was included, and we confirmed that the administration of RTX in older patients with IMN should be managed by age.

In this study, our data showed that higher-dose RTX regimen results in more adverse events in older patients with IMN. Moreover, the results of multivariate logistic regression analysis suggest that both age and different treatment regimens were correlated with infection. Further results of regression analysis for patients aged ≥60 years suggested that the risk of infection in patients was associated with different treatment regimens. Because ALB is low, patients with IMN are at higher risk of infection. Elderly IMN patients are at a higher risk of infection due to the aging state. RTX causes B cell depletion, leading to a further increase in the risk of infection. Therefore, RTX should be used more cautiously in elderly patients with IMN. This study confirms that a low-dose, long-course RTX regimen should be recommended for patients with IMN aged ≥60 years.

Additionally, some studies suggested that the number of CD20^+^/CD19^+^ B lymphocytes is not only one of the important indicators to judge the therapeutic effect of RTX, but also one of the important indicators to predict the adverse reactions of RTX in IMN patients [16,17]. Generally, a lower number of B lymphocytes predicting a higher incidence of adverse reactions in IMN patients after RTX was used [16,17]. Due to the difference in physical fitness between the young and the aged, elderly patients with IMN have a higher risk of infection when the peripheral blood CD19^+^ B lymphocyte count is decreased compared to the young. Not only that, in IMN patients who respond effectively after RTX, the number of CD19^+^ B lymphocytes will all drop to 0, but not all patients will develop infections. This means that using the number of CD19^+^ B lymphocytes as an indicator to predict the occurrence of infections in IMN patients after RTX lacks sensitivity and specificity. Our data showed that the percentage of CD19^+^ B lymphocyte did not predict the incidence of infection after RTX used in IMN patients. Nevertheless, in the first month after RTX was used, compared with the percentage of CD19^+^ B lymphocytes, the decreased percentage of eosinophilic granulocytes was more sensitive in predicting the incidence of infection in elderly patients with IMN, which need to be verified by a lager random study.

This study has several limitations. First, as a single-center retrospective study, the potential for selection bias and confounding factors cannot be fully excluded. Second, the sample size in this study is small, which may cause certain bias to the results. Finally, the follow-up period of this study was 9 months, which was relatively short. Thus, a retrospective cohort study with a larger sample and a longer follow-up period is needed to confirm the conclusion of this article.

## 5. Conclusions

The use of RTX in patients with PLA2R-associated IMN should be administered by age. The low-dose and long-course RTX regimen should be recommended for elderly patients with IMN, and the percentage of eosinophilic granulocytes is a better risk predictor of infections after 1 month RTX used in older patients with IMN compared to the percentage of CD19^+^ B lymphocytes.

## Data Availability

The data underlying this article will be shared on reasonable request to the corresponding author.
